# Fine Tuning of the Arrangement of Non-Close-Packed Structures: Specific Ion Effects of Lanthanide Cations

**DOI:** 10.1021/acsomega.5c13357

**Published:** 2026-03-16

**Authors:** Melike Barak, Paolo Scharmann, Christina Graf

**Affiliations:** † Department of Chemical Engineering and Biotechnology, Darmstadt University of Applied Sciences, Stephanstr. 7, 64295 Darmstadt, Germany; ‡ Eduard-Zintl-Institute of Inorganic and Physical Chemistry, Technical University of Darmstadt, Peter-Grünberg-Street 12, 64287 Darmstadt, Germany; § EUt+ Institute of Nanomaterials & Nanotechnologies EUTINN, European University of Technology, European Union, https://www.univ-tech.eu/eutinn

## Abstract

Specific ion effects are widespread and play a crucial role in many fields across chemical and biological systems. Here, the identity of the constituent ions is a determining factor, not just their charge or concentration. Therefore, these effects show a complex dependence on properties such as ion polarizability, size, and hydration shell. The formation of non-close-packed structures (NCPS) of colloidal particles on gold substrates in the presence of different ions enables the investigation of ion effects by examining particle arrangement. Due to their chemical similarity and common valence, lanthanide (Ln^3+^) ions were explored to determine the effect of polarizability exclusively. It is shown that the tendency of particles to aggregate correlates with the polarizability of the Ln^3+^ ions. Colloidal stability was increased in the presence of highly polarizable ions (La^3+^), whereas a less polarizable ions (Lu^3+^) led to the promotion of aggregation. Contrary, the nearest neighbor distances of the colloidal particles remain approximately constant through all lanthanide ions. Moreover, it could be shown that in the presence of Ln^3+^ ions, the maximum surface coverage of particles is consistently lower than for monovalent ions or in the random sequential adsorption (RSA) model because multivalent co-ions induce long–range interactions. Therefore, using trivalent co-ions allows for adjustment of interparticle distances over a larger range than monovalent ions. The maximum surface coverage of particles at ionic strengths from 0.1 to 1 mM decreases with increasing ion valency. A maximum surface coverage of 0.43 is achieved with GdCl_3_ at an ionic strength of 300 mM.

## Graphical abstract

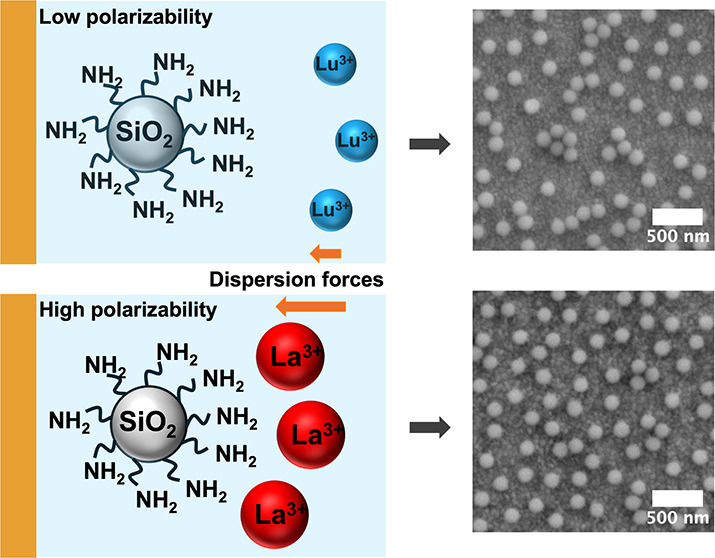

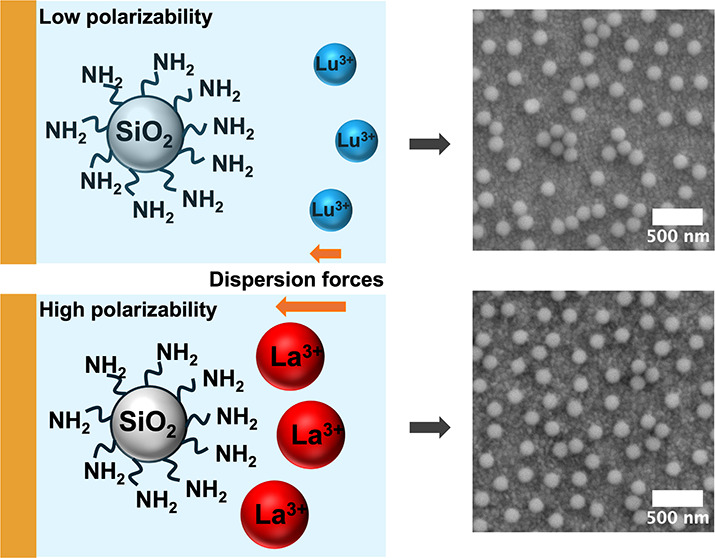

## Introduction

Specific ion effects are a fundamental phenomenon observed in many disciplines, including biology, chemistry, physics, materials science, colloid and interface science.^1–7^ It describes the influence of ions not only through their charge, but also their unique chemical identities. Hofmeister carried out the pioneering study on specific ion effects, establishing the foundation of the Hofmeister series, which orders salts based on their ability to salt in or out egg protein.^8^ The specific ion effect is a broader term than the Hofmeister series, encompassing interactions of ions with particles^9–11^ interfaces,^12–14^ and macromolecules^15,16^ controlled by parameters such as polarizability, hydration, ion size, and dispersion forces.^17^ Specific ion effects govern solubility, stability, aggregation, and self-assembly of particles, polymers, and proteins by changing nonelectrostatic forces.^18,19^

Colloidal self-assembly is a well-known nanofabrication method that directly transfers particles from a dispersion onto a surface. In this method, interactions between particles, e.g., van der Waals attraction and electrostatic repulsion, and between particles and substrate, e.g., van der Waals attraction, electrostatic interactions, and capillary forces, are critical for forming monolayers.^20^ The fabrication of non-close-packed structures (NCPS) through self-assembly is achievable; however, it still often requires a multistep process and can be tedious due to the requirement for a template or selective removal.^21–23^

Schmudde et al. reported a direct method for preparing NCPS by colloidal self-assembly of charged particles using an orbital shaker or a quartz crystal microbalance.^24,25^ The arrangement of particles here is determined by the interplay between interparticle electrostatic repulsion and the interaction between the substrate and particles, including particle and surface roughness. Interparticle distances, the extent of particle aggregation, and surface coverage can be further controlled by varying the ionic strength of an added electrolyte.^25^ In our previous study, we examined the effect of specific ions and ion valency on the formation of NCPS. Particles dispersed in aqueous solutions of salts with different ionic strengths, consisting of different co- and counterions chosen from the Hofmeister series, were deposited by colloidal self-assembly on gold-coated substrates. Due to electrostatic repulsion between charged particles and slow drying, particles assembled into non-closed-packed monolayers. Aggregation behavior and nearest neighbor distances were investigated in detail on the dried substrates.^26^ In contrast to previous studies, which examined the ion effect by determining the critical coagulation concentration (CCC) using time-resolved dynamic light scattering (DLS), this approach enables investigation of ion-dependent aggregation at low ionic strengths, thereby allowing characterization at the early onset of aggregation. Specific ion effects are explained in terms of the polarizability of ions in aqueous solutions by Ninham et al.^6,17,27^ and the polarity of the ion and the surface by López-León et al.^5^ The Schulze-Hardy rule demonstrates how the valence of ions affects particle behavior.^28^

Lanthanide (Ln^3+^) ions exhibit distinct behavior: their ionic radii decrease with increasing atomic number, even as the number of 4f electrons increases across the series, a phenomenon known as the lanthanide contraction. This occurs because the 4f electrons shield the nuclear charge poorly, leading to an enhancement of the effective nuclear charge.^29,30^ The lanthanide series is not included in the Hofmeister series; nevertheless, some studies have examined the influence of La^3+^ ions along with other ions from the Hofmeister series. Fries et al. studied protein–protein and protein–surface interactions using La^3+^ ions.^31^ La^3+^ ions were selected to investigate the low-frequency dynamics of water molecules in the presence of kosmotropic cations by observing the water structure, extending the study beyond the classical Hofmeister series by González-Jiménez et al.^32^ Moreover, the specific ion effect, typically assessed at high electrolyte concentrations (≥100 mM)^17^ via critical coagulation concentration measurements, cannot be studied with lanthanide salts due to their incomplete dissociation under these conditions.^33^ Rudolph and Irmer reported that for LaCl_3_, no complex formation was observed at concentrations below 0.1 M,^34^ and for LuCl_3_, extrapolation of the Raman data indicates that concentrations of 0.5 M and lower, the Lu^3+^ cation exists predominantly in a fully hydrated form.^35^

The co- and counterions from the Hofmeister series were ranked independently of their valence and size. In the present study, we chose ions from the lanthanide series due to their similar chemical properties and common ion valencies. Using these ions allows us to examine the effect of ions solely as a function of polarizability while keeping the valence constant. Furthermore, we previously reported that particle aggregation decreased with increasing ion valence, consistent with the inverse Schulze–Hardy rule.^26^ Based on this observation, trivalent ions were selected in order to examine their influence on the NCPS more comprehensively. Here, NCPS from amino-functionalized silica particles dispersed in aqueous solutions of various LnCl_3_ with different ionic strengths were prepared on gold substrates. Using low ionic strength ranging from 0.1 mM to 6 mM ensured complete dissociation of the lanthanide salts. The NCPS were characterized by field-emission scanning electron microscopy (FESEM) after controlled drying. The influence of lanthanide ions was evaluated by analyzing nearest neighbor distances, aggregation behavior, and surface coverage from FESEM images.

## Experimental Section

### Materials

Cyclohexane (Supelco, 99.5%), l-arginine (Sigma-Aldrich, ≥98%), ammonia solution (Sigma-Aldrich, 25% wt. in water), tetraethyl orthosilicate (TEOS, Sigma-Aldrich, 98%), ethanol (Supelco, absolute), *N*-(6-aminohexyl)­aminopropyltrimethoxysilane (AHAPS, Fluorochem, 92%), and sodium dodecyl sulfate (SDS, Sigma-Aldrich, >99%) were used as received without further purification. The following analytical grade salts were acquired: NaCl (>99.5%), LaCl_3_·7H_2_O (>99%), SmCl_3_·6H_2_O (>99%), GdCl_3_·6H_2_O (>99.999%), DyCl_3_·6H_2_O (>99.9%), ErCl_3_·6H_2_O (>99.9%), TmCl_3_·6H_2_O (>99.99%), YbCl_3_·H_2_O (>99.9%), LuCl_3_·6H_2_O (>99.99%) from Sigma-Aldrich, PrCl_3_·6H_2_O (>99.95%) from Thermo Scientific, and MgCl_2_ (≥99%) from Roth. Ultrapure water (resistivity of 18.2 MΩ cm) was obtained using a Purelab Flex 2 system (ELGA). An orbital shaker (VXR Vibrax) from IKA was used for particle assembly on the substrates. Glass slides (1 cm^2^) coated with a 200 nm gold layer were purchased from Ssens B.V., Netherlands.

### Preparation of Silica Particle Dispersions in Salt Solutions and NCPS

Amino-functionalized silica particles were dispersed in aqueous salt solutions at a concentration of 0.1 g/L. The salt solutions were prepared with ionic strengths of 0.1, 0.5, 1, 3, 6, 10, 50, and 300 mM using ultrapure water (0.055 μS/cm). Silica particles in ethanol were initially dispersed for 15 min using an ultrasonic bath (Sonorex Super RK 512 H, Bandelin Electronic), followed by 4 min of sonication using a sonotrode (SPX150, Branson) equipped with a 3.2 mm tip. The prepared particle dispersions in salt solution were further sonicated in an ultrasonic bath for 15 min. To generate NCPS, the particles were transferred onto the gold surface using an orbital shaker (see Supporting Information for details).

### Characterization

A Zetasizer Nano ZS (Malvern Instruments Ltd.) was used to measure the hydrodynamic diameter and the zeta potential. The particle distribution of non-close-packed ordered silica particles, as well as the particle size were analyzed using a Hitachi SU-5000 Schottky Field Emission Scanning Electron Microscope (FESEM) with an accelerating voltage of 5 kV. For each experiment, six different spots on the gold substrate were selected to obtain an overview, and the resulting FESEM images were analyzed. Nearest neighbor distances (center-to-center distances) were measured using the FIJI program with the Simple Analysis 2D/3D plugin. The roughness of particles and substrates was determined using transmission electron microscopy (TEM; Zeiss EM109, 80 kV) and atomic force microscopy (AFM; Cypher, Asylum Research, Oxford Instruments, Santa Barbara, USA) with a PPP FM-AuD10 cantilever (Nanosensors, Neuchatel, Switzerland).

## Results and Discussion

Rough (*R*_q_: 1.2 ± 0.15 nm) amino-functionalized silica particles with a diameter of 148 ± 5 nm were synthesized by a modified Hartlen method.^24,36^ *N*-(6-aminohexyl)­aminopropyltrimethoxysilane (AHAPS) was used to functionalize the surface of particles based on refs 26 and 37 which converted the surface charge from negative to positive, resulting in a zeta potential of 43 ± 1 mV in water. The particle distribution and aggregation behavior of the amino-functionalized rough silica particles were investigated in aqueous LnCl_3_ solutions by analyzing their self-assembly into a non-close-packed monolayer on gold substrates, as previously described for the effect of ions from the Hofmeister series.^26^ In this study, the counterion (chloride) remains unchanged while the influence of the lanthanide ions acting as co-ions was measured. The ionic strength of LnCl_3_ was mainly varied from 0.1 to 6 mM. For this purpose, particle dispersions in salt solutions were deposited on gold-coated glass substrate (*R*_q_: 1.62 ± 0.02 nm) using an orbital shaker as described by Schmudde et al.^25^ At this stage, the particles bind to the gold substrate due to the affinity between the amino groups and the gold surface. Subsequently, the substrates were rinsed with water and ethanol and allowed to dry slowly, resulting in the formation of non-close-packed structures (NCPS). These structures were analyzed using FESEM.

Before transferring particles onto gold substrates, the hydrodynamic diameter and zeta potential of particles dispersed in salt solutions were analyzed using dynamic light scattering (DLS) and electrophoretic light scattering, respectively, to evaluate their colloidal stability. The hydrodynamic diameter and zeta potential of particles in aqueous LnCl_3_ solutions measured at ionic strengths of 3 and 6 mM are listed in Table S1 (see Supporting Information). The two highest ionic strengths were selected because no significant changes in hydrodynamic diameter or zeta potential were observed at lower ionic strengths. These results are consistent with those of our previous study.^26^ The hydrodynamic diameter provides insights into the aggregation behaviors of particles in dispersion. It is generally larger than the dried particle size, as measured by FESEM or TEM, because DLS measures particle size by accounting for particle motion and the surrounding solvation layer. However, a significantly larger hydrodynamic diameter compared to FESEM diameter indicates the presence of aggregates in the dispersion. The average hydrodynamic diameters of the particles dispersed in LnCl_3_ solutions with different ionic strengths were similar, suggesting that no aggregates were present, as observed for most salt solutions of mono- and divalent cations at similar ionic strength studied in our previous work.^26^ The zeta potential of the particles was at least 55 mV, which implies high colloidal stability since zeta potential values above ±40 mV indicate electrostatic stability of the particles.^38^

### Ionic Strength–Dependent Particle Distribution on Gold Surfaces in the Presence of LaCl_3_ and LuCl_3_

Figure 1 shows FESEM images of amino-functionalized silica particles dried from LaCl_3_ and LuCl_3_ solutions with ionic strengths ranging from 0.1 mM to 6 mM deposited on gold substrates, along with their corresponding histogram graphs. For this comparison, the first and last elements of the lanthanide series were chosen to visualize the most different ionic behavior within this series. FESEM images were used to analyze nearest neighbor distances, yielding corresponding histograms. The histograms show two distinct distributions: the left one represents aggregates, while the right one corresponds to particles with controlled distances. The mean distance of the left peak in the distribution of the nearest neighbor graph was defined as the distance at which particles are in contact, i.e., aggregated. In both salt solutions, the intensity of the aggregation distribution peaks increases with ionic strength, since a higher ionic strength diminishes repulsive forces between particles, shortens the Debye length, and consequently promotes aggregation. At the same time, the average nearest neighbor distance of the nonaggregated particles is reduced with the compression of the Debye length, allowing particles to approach each other more closely. A shift of the controlled-distance peaks to lower values is clearly seen in the histogram graphs as the ionic strength increases. The NCPS prepared with aqueous LaCl_3_ and LuCl_3_ solutions at an ionic strength of 0.1 mM exhibit almost no aggregation (≈1%), as shown in Figure 1a,b,k, l. At an ionic strength of 6 mM, the highest percentage of aggregates was found in the NCPS (see Figure 1i,j,s,t). The histogram graphs show that for all ionic strengths, the percentage of aggregates in the NCPS prepared from LaCl_3_ is lower than in those prepared from LuCl_3_. In the NCPS prepared from LaCl_3_, the histograms show a broader distribution of the controlled interparticle distances than in those prepared from LuCl_3_ at an ionic strength of 1 mM, whereas the opposite trend was observed for the distribution of the aggregates (see Figure 1,p). In the NCPS prepared from LuCl_3_, the intensity of the peak corresponding to controlled interparticle distances is lower than in those prepared with LaCl_3_ (see Figure 1h,j,r,t). Although the FESEM images did not distinctly show the influence of the salts on NCPS formation, the corresponding histograms reveal that LuCl_3_ enhances particle aggregation more strongly than LaCl_3_.

**Figure 1:**
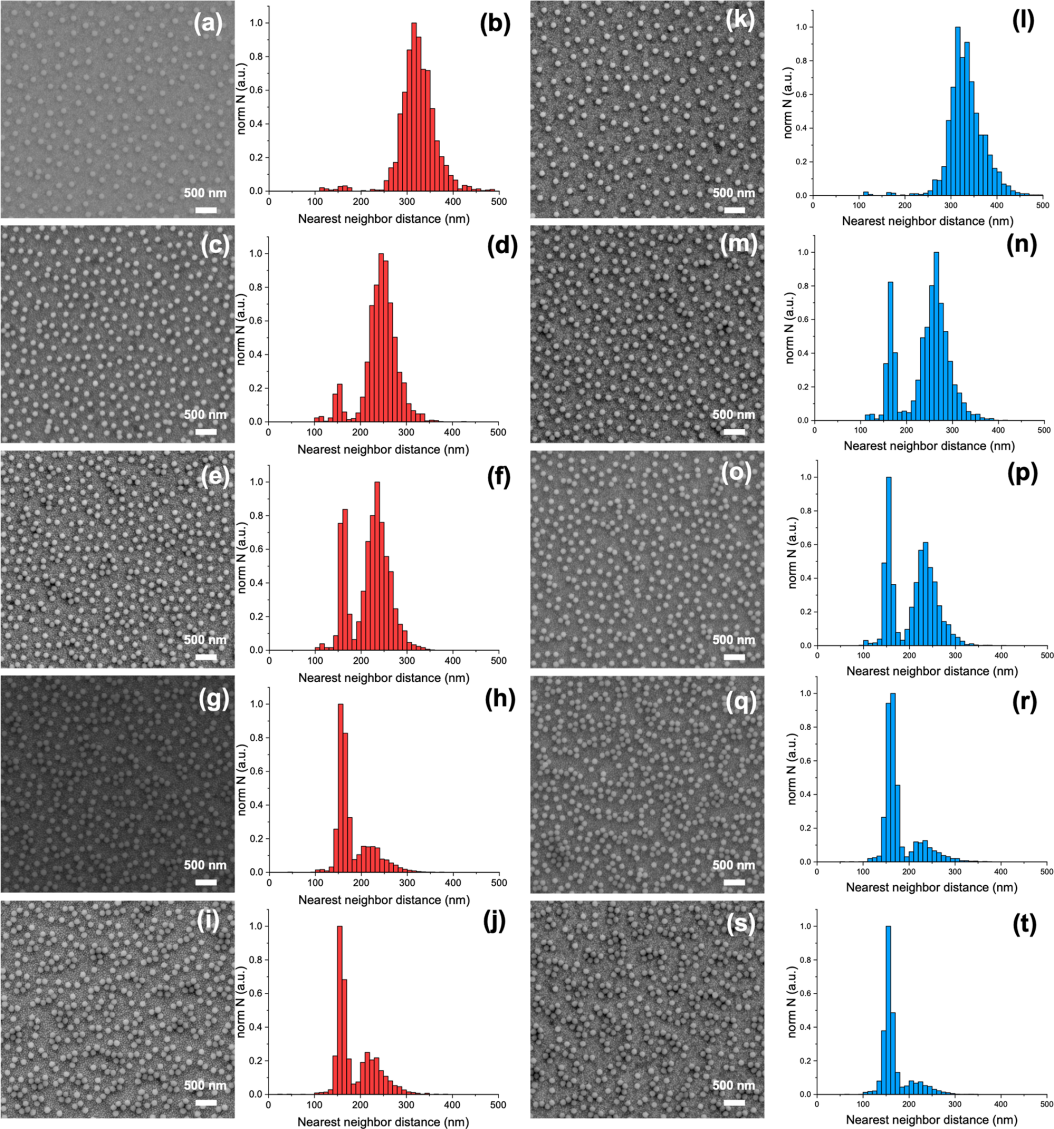
FESEM images and corresponding histograms which show particle distribution of amino-functionalized silica particles obtained by drying their aqueous dispersion with LaCl_3_ (red) at ionic strengths of 0.1 mM (a,b), 0.5 mM (c,d), 1 mM (e,f), 3 mM (g,h) and 6 mM (i,j) and LuCl_3_ (blue) at ionic strength of 0.1 mM (k,l), 0.5 mM (m,n), 1 mM (o,p), 3 mM (q,r) and 6 mM (s,t).

### Influence of Lanthanide Ions on the Aggregation of Particles and the Interparticle Distances

The aggregation behavior of the amino-functionalized particles deposited on the gold substrates was investigated as a function of the type of lanthanide ions present at varying ionic strengths, as depicted in Figure 2. The order of the ions in this figure represents the decrease in ionic radius and the corresponding decrease in the polarizability from La^3+^ to Lu^3+^, and was taken from a study by Shannon.^30^

**Figure 2:**
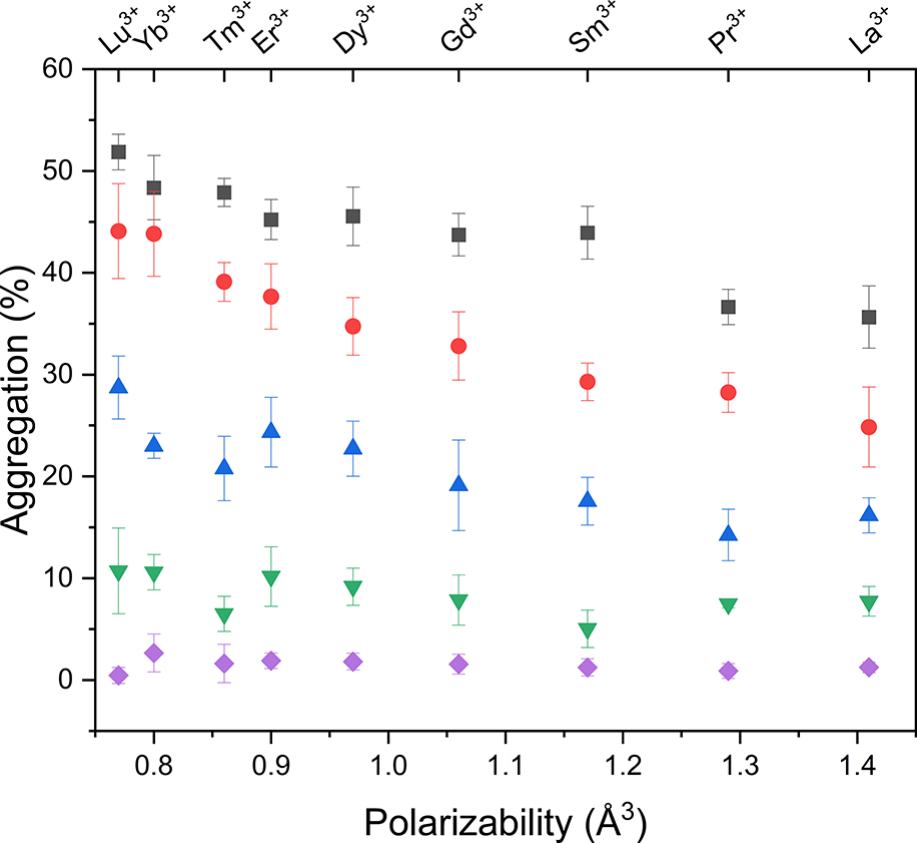
Aggregation percentages of amino-functionalized silica particles on gold substrates prepared in the presence of different LnCl_3_ solutions versus the position of the corresponding lanthanide ions based on their polarizabilities. (The values for the polarizabilities are taken from Shannon.^30^) The ionic strengths are 6 mM (black squares), 3 mM (red circles), 1 mM (blue triangles), 0.5 mM (green inverted triangle), and 0.1 mM (purple quadrangles).

Increasing the ionic strength weakens electrostatic repulsion between particles, thereby increasing the number of aggregates on the NCPS on the gold substrates. At low ionic strength values (0.1 and 0.5 mM), the aggregation percentages are nearly identical for all lanthanide ions. However, at higher ionic strengths (1, 3, and 6 mM), the aggregation percentage decreases significantly with increasing polarizability (see Figure 2). This can be explained by their higher polarizability, since La^3+^ ions induced lower aggregation than Lu^3+^ ions. López-León et al. compared the influence of co-ions by comparing three different monovalent anions with different polarizabilities but also significantly different chemical properties on the critical coagulation concentration of negatively charged colloidal particles. According to their theory, the local charge density at the surface proximity increases due to the accumulation of more polarizable ions (stronger dispersion forces), which in turn promotes higher colloidal stability than in systems with less polarizable ions.^5^ In our study, we extended this theory for polarizable trivalent cations on positively charged particle surfaces. Due to their chemical similarity and identical ion valencies, lanthanide ions were chosen to demonstrate the effect of polarizability. The present results suggest that an increased approach of more polarizable cations can strengthen interparticle repulsion by enhancing local charge density, resulting in less aggregation on the gold substrates. Surprisingly, however, the hydrodynamic diameters and zeta potentials of particles in the presence of different LnCl_3_ are similar and independent of the polarizability of the lanthanide ions (see Table S1 in the Supporting Information). This may be explained by considering that the zeta potential is the potential at the slipping plane, reflecting the situation there, while the interaction of the lanthanide ions with silica particles takes place closer to the particle surface and is therefore not adequately detected by DLS and electrophoretic light-scattering measurements in dispersion. Several studies have examined the effect of multivalent co-ions on surface charge densities. Ruiz-Cabello et al.^39^ claimed that multivalent co-ions do not influence the surface charge of colloidal particles, and that only multivalent counterions affect the surface charge due to ion adsorption on the surface. On the contrary, Rosenholm et al.^40^ stated that multivalent co-ions can adsorb onto particle surfaces, thereby altering the surface potential of the particles. The present results suggest that at constant ion valency, ion polarizability is an important factor influencing the aggregation behavior of colloidal particles.

Figure 3 shows the dependence of the inverse square root of ionic strength, which is directly proportional to the Debye length (κ^–1^) on the nearest neighbor distances of dried silica particle arrays prepared in the presence of different LnCl_3_ solutions. Nearest neighbor distances were determined, excluding aggregated particles from the analysis. As the ionic strength increases from 0.1 mM to 6 mM, the nearest neighbor distances decrease linearly for each LnCl_3,_ consistent with an increase in surface coverage, as discussed in detail below.^25^ Increasing ionic strength reduces electrostatic repulsion between particles in dispersion and enhances electrostatic screening, thereby reducing the Debye length.^41^ As a result, attractive van der Waals forces between particles strengthen, causing them to move closer together. Similar trends in nearest neighbor distances were observed in the study by Schmudde et al.,^25^ which investigated the influence of the ionic strength on NaCl solutions, as well as in our previous research, which studied the impact of Hofmeister series ions and their ionic strengths.^26^ In contrast to the aggregation percentages, the nearest neighbor distances are similar for all LnCl_3_ at any given ionic strength. This suggests that the polarizability of the ions does not influence the range of the electrostatic repulsion and, consequently, does not affect the thickness of the double layer. This finding is in line with the observation that the zeta potential and the hydrodynamic diameter are also independent of the nature of the lanthanide ion (see above). Thus, the experiments demonstrate two distinct effects. The polarizability of the Ln^3+^ ions governs the aggregation behavior. In contrast, the ionic strength primarily determines the interparticle spacing.

**Figure 3:**
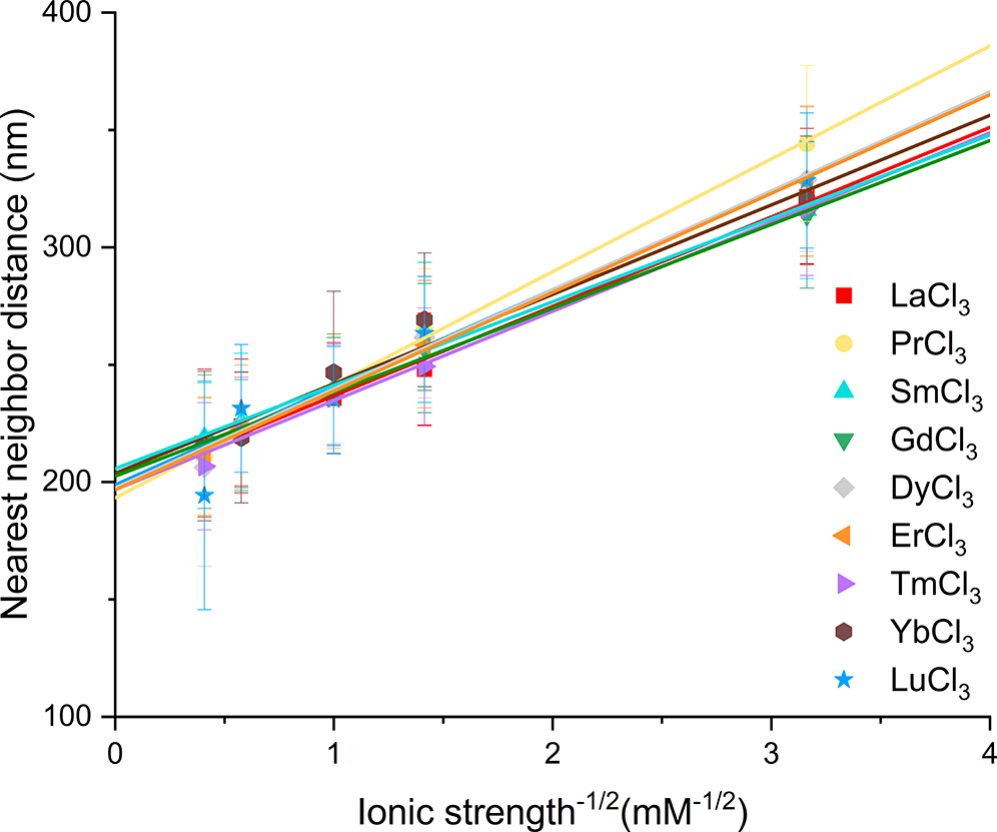
Nearest neighbor distances of the amino-functionalized silica particles on gold substrates versus the inverse square root of the ionic strength for LnCl_3_.

### Surface Coverage Behavior of Particles

The maximum surface coverage of the particles was plotted as a function of the dimensionless screening parameter (κa, where κ is the inverse Debye length and a is the particle radius) for LnCl_3_, NaCl, and MgCl_2_, as shown in Figure 4a. The particles were randomly adsorbed onto the surfaces, without superimposing on previously adsorbed particles. Therefore, a random sequential adsorption (RSA) model was applied to analyze the data. In this model, the particles adsorb onto the surface until they reach the jamming limit, which is 0.547 for monodispersed hard disks.^42,43^ According to this, the maximum surface coverage is primarily controlled by the electrostatic repulsive forces between particles, which are determined by the ionic strength of the particle dispersion. As the ionic strength decreases, and consequently the dimensionless screening parameter κa diminishes, the maximum surface coverage declines. Semmler et al. developed an effective hard-sphere model with charge saturation.^44^ Plots taken from this study for particle radii of 20 and 100 nm are shown in Figure 4 a (solid lines). In previous studies, symmetric monovalent electrolytes, e.g., NaCl^44–46^ and KCl^47,48^ were used at varying ionic strengths. The authors of these studies reported that the maximum surface coverages are consistently higher than those predicted by the simple theory. However, the deviations from the RSA model observed in the present study are significantly larger than those previously reported for monovalent electrolytes. The values obtained for silica particles dried in the presence of LnCl_3_ are even lower than those obtained for particles with significantly larger diameters. To examine differences depending on the ion valence, additional experiments were conducted using NaCl and MgCl_2_ at ionic strengths of 0.1, 0.5, and 1 mM. As a result, the maximum surface coverage was found to decrease in the order: NaCl > MgCl_2_ > LnCl_3_ (see Figure 4a). Ruiz-Cabello et al. studied the effect of multivalent co-ions on force curves and found that these ions are more strongly excluded from the region between particle surfaces compared to monovalent co-ions. As a result, the double-layer repulsive force profile between particles becomes softer and extends to larger distances.^39^ In our study, we observed a similar trend, in which Ln^3+^ ions increase the spacing between particles, resulting in larger nearest neighbor distance values compared to Na^+^ and Mg^2+^ (see Supporting Information in Table S2), and consequently, the surface coverage is the lowest in the presence of the lanthanide ions. Figure 4b displays the variation of the radial distribution function with center-to-center distance for NaCl, MgCl_2_, and LaCl_3_. The second peak, corresponding to the nearest neighbor distance, shifts to higher values with increasing ion valence. To investigate how the maximum surface coverage changes at high ionic strengths, experiments were conducted with GdCl_3_ at higher ionic strengths up to 600 mM, Gd^3+^ was selected because of its middle position in the lanthanide series. The maximum surface coverage was 0.43 at an ionic strength of 300 mM (see Figure 4a). Figure 5 shows representative FESEM images of particle arrays dried from GdCl_3_ solutions at ionic strengths ranging from 0.1 mM to 300 mM. The surface coverage of the NCPS and the aggregation of the particles increase with the ionic strength and reaches its maximum at 300 mM. Above this ionic strength, the loss of colloidal stability leads to particle aggregation in the dispersion (The hydrodynamic diameter at 600 mM was measured as 502 ± 44 nm, which is significantly larger than the size measured in FESEM, indicating colloidal instability.), which in turn reduces the number of particles adsorbed on the surface and consequently decreases the surface coverage (see Figure S1 in the Supporting Information). Therefore, it is essential that particles remain colloidally stable in dispersion to perform colloidal self-assembly while preventing coagulation. In this study, we measured the hydrodynamic diameter of particles prepared in NaCl, MgCl_2_, and GdCl_3_ solutions at ionic strengths ranging from 0.1 to 6 mM (see Table S3 in the Supporting Information) to evaluate particle stability. At an ionic strength of 3 mM, particles prepared in NaCl solution show a slight increase in hydrodynamic diameter (190 ± 2.0 nm) compared to those prepared in MgCl_2_ and GdCl_3_ solutions, indicating beginning aggregation. At 6 mM ionic strength, particles prepared in NaCl and MgCl_2_ solutions exhibited hydrodynamic diameters of 250 ± 2 nm and 258 ± 5 nm, respectively. In contrast, particles prepared in a GdCl_3_ solution showed a smaller hydrodynamic diameter of 165 ± 2 nm. Furthermore, particles dispersed in the NaCl solution with an ionic strength of 50 mM exhibited a hydrodynamic diameter of 711 ± 62 nm, whereas those prepared in the GdCl_3_ solution at the same ionic strength showed a significantly smaller hydrodynamic diameter of 174 ± 0.5 nm. This is because positively charged silica particles dispersed in solutions of trivalent ions are more stable than in solutions of monovalent and divalent ions at the same ionic strength, a behavior that is consistent with the inverse Schulze–Hardy rule,^28,49^ which predicts an increase in the critical coagulation ionic strength (CCIS) with increasing ionic valence. Trivalent co-ions induce large interparticle distances at the same ionic strength and provide higher colloidal stability at high ionic strengths compared to monovalent and divalent ions; therefore, trivalent cations enable tuning of interparticle distances in nanoparticle arrays in a wide range from 344 ± 33 to 194 ± 49 nm. In conclusion, the present data show that the maximum surface coverage at a given ionic strength decreases with the increasing valency of the co-ions. This deviation from RSA can be explained by the longer-range soft repulsion induced by multivalent co-ions compared to that of monovalent ones, and goes along with the higher colloidal stability of the particles in dispersions of co-ions with a higher valency.

**Figure 4:**
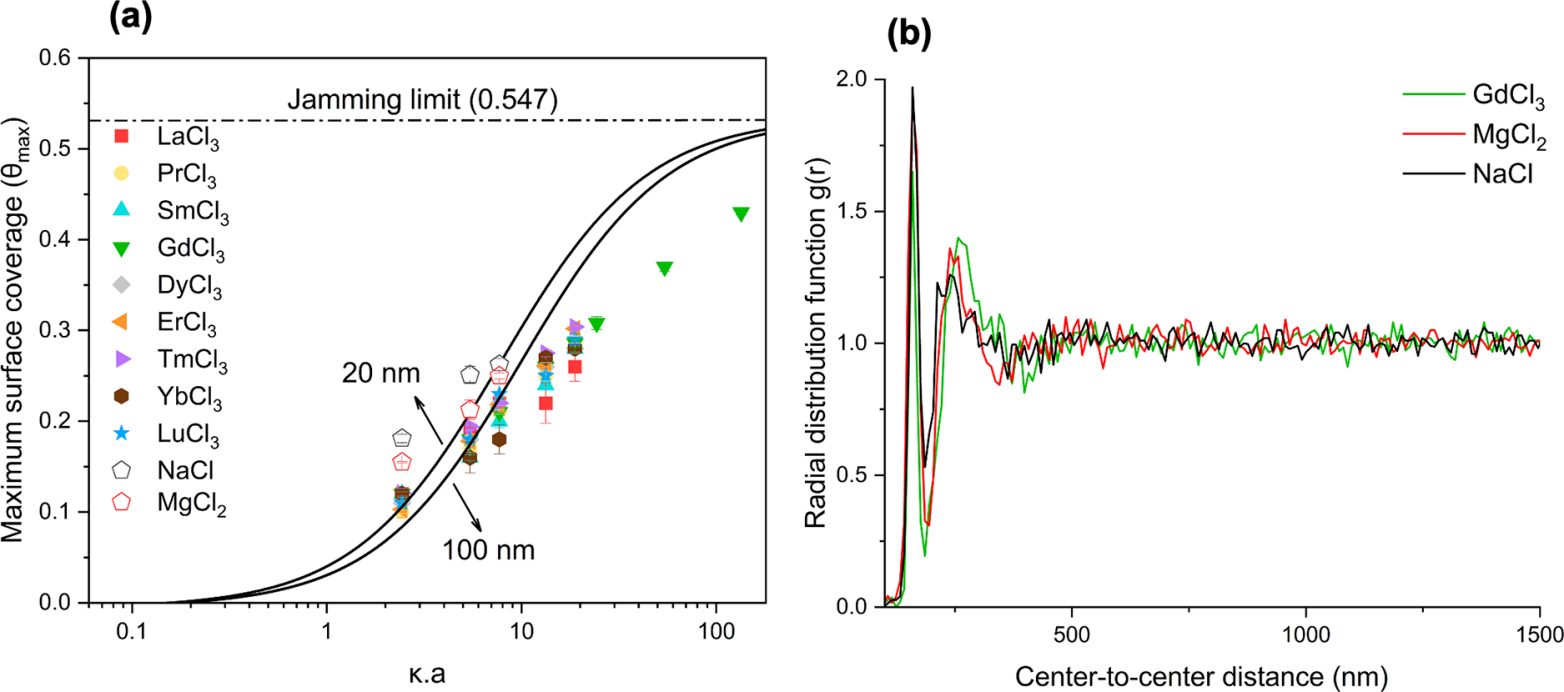
Maximum surface coverage of amino-functionalized silica particles dispersed in aqueous LnCl_3_, NaCl, and MgCl_2_ solutions on gold substrate against the dimensionless screening parameter κa, where a is the particle radius, and κ is the inverse Debye length (a). The solid lines adopted from Semmler et al.,^44^ [Semmler, M.; Mann, E. K.; Rička, J.; Borkovec, M. Diffusional Deposition of Charged Latex Particles on Water–Solid Interfaces at Low Ionic Strength. Langmuir 1998, 14 (18), 5127–5132. Copyright 1998 American Chemical Society.], represent calculations based on an effective hard-sphere model with charge saturation. Radial distribution function *g*(*r*) of amino-functionalized silica particles on gold substrates prepared with GdCl_3_, MgCl_2_, and NaCl solutions at an ionic strength of 1 mM as a function of the center-to-center distance (b).

**Figure 5:**
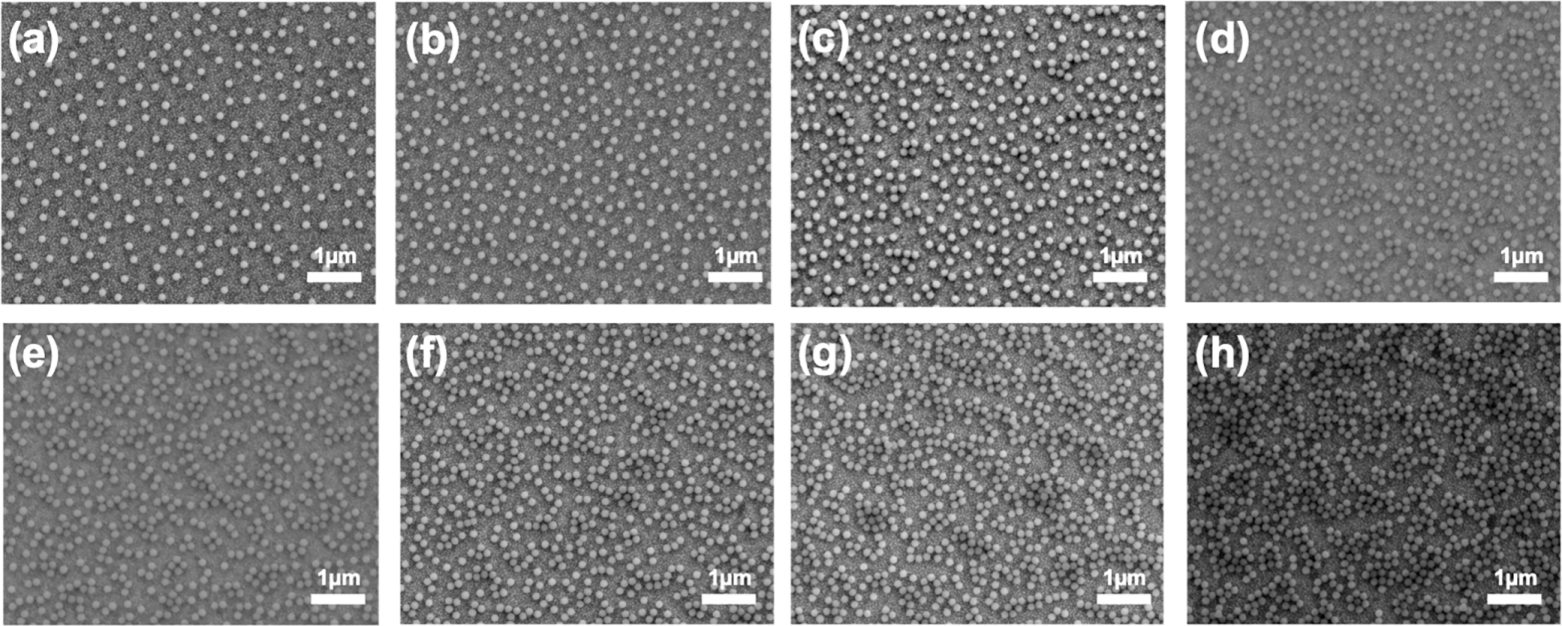
FESEM images showing the dried NCPS prepared from the amino-functionalized silica particles dispersed in GdCl_3_ solutions at ionic strengths of 0.1 mM (a), 0.5 mM (b), 1 mM (c), 3 mM (d), 6 mM (e), 10 mM (f), 50 mM (g), and 300 mM (h).

## Conclusions

Lanthanide ions, which are chemically similar but significantly change their radius throughout their series due to the lanthanide contraction, provide an ideal system for investigating the influence of co-ion polarizability while maintaining a constant valence. For this reason, we extended our previously reported straightforward and sensitive method for studying the effects of specific ions and ion valency on particle arrangement during the formation of non-close-packed structures using ions of the lanthanide series. This approach eliminates the complexity associated with interpreting the effects of ions in the Hofmeister series, which does not rank ions solely by size or valence.

The effects of Ln^3+^ ions on the arrangement of non-close-packed ordered structures of positively charged silica particles on gold substrates are investigated, focusing on adjustments in interparticle distance, aggregation behavior, and maximum surface coverage. The higher the polarizability of the lanthanide ions, the fewer aggregates were found in the dried, non-close-packed arrays. Regardless of this effect, the nearest neighbor-distance in the dried arrays was almost the same for all investigated Ln^3+^ ions at a given ionic strength, as well the hydrodynamic diameter and zeta potential of the particles in dispersion were not influenced by the nature of the lanthanide ions. This indicates that the polarizability of the Ln^3+^ ions controls the aggregation behavior, while the ionic strength primarily determines the interparticle spacing in the dried state and in dispersion.

The maximum surface coverage was achieved at an ionic strength of 300 mM, as exemplarily shown for GdCl_3_. A comparison with NaCl and MgCl_2_ shows that the surface coverage decreases with increasing ionic valence, indicating that trivalent ions induce a less closely packed ordering of colloidal particles on the substrates. This phenomenon can be explained by the fact that multivalent ions induce longer-range double-layer forces. In line with this finding, the nearest neighbor distance decreased with decreasing ion valence. By providing both increased interparticle distances at the same ionic strengths and enhanced colloidal stability at high ionic strengths, trivalent co-ions enable wide-ranging control of nanoparticle array spacing compared to ions with lower valency.

## Supplementary Material



## Notes

Additional experimental details, zeta potential and dynamic light scattering data of amino-functionalized silica particles in different salt solutions, nearest neighbor distance distances of these particles in non-closed-packed arrays, and additional scanning electron microscopy data (PDF)
