# Supplementary material for: Fine Tuning of the Arrangement of Non-Close-Packed Structures: Specific Ion Effects of Lanthanide Cations
Source: ACS Omega. 2026 Mar 16;11(12):19579–87. doi: 10.1021/acsomega.5c13357 (PMC13044851; doi:10.1021/acsomega.5c13357)
Supplement: Supplementary file 1 [file ao5c13357_si_001.pdf]

Fine Tuning of the Arrangement of Non-Close-Packed Structures: Specific Ion Effects of Lanthanide Cations

Melike Barak[†‡§], *Paolo Scharmann*[†§], *and Christina Graf*[†§]*

AUTHOR ADDRESS.

† Department of Chemical Engineering and Biotechnology, Darmstadt University of Applied Sciences, Stephanstr. 7, 64295, Darmstadt, Germany

‡ Eduard-Zintl-Institute of Inorganic and Physical Chemistry, Technical University of Darmstadt, Peter-Grünberg-Str. 12, 64287, Darmstadt, Germany

§ EUt+ Institute of Nanomaterials and Nanotechnologies, EUTINN, European University of Technology, European Union, https://www.univtech.eu/eutinn

1. Synthesis of Amino-Functionalized Rough Silica Particles.

Silica particles with a diameter of 148 ± 5 nm were synthesized using a multistep growth process reported in our previous work.[1,2] Subsequently, the particles were functionalized with N-(6-aminohexyl)aminopropyltrimethoxysilane (AHAPS) under an argon atmosphere as described in ref.[3,4] The amino-functionalized silica particles were dispersed in ethanol and stored under argon to prevent the introduction of CO_2.

2. Preparation of Non-Close-Packed Ordered Silica Particles.

Non-close-packed ordered silica particles were prepared according to previous reports.[1,5] Briefly, gold-coated glass substrates were cleaned with 2 % SDS solution, followed by UV-ozone (PSD Pro series, Novascan) treatment. The substrates were placed in centrifuge tubes (50 mL) containing the particle dispersion prepared with the salt solution and agitated for 1 h at 600 rpm using an orbital shaker (VXR Vibrax, IKA). Afterward, the substrates were rinsed twice with water and once with ethanol, then dried slowly for 16 h at room temperature in loosely capped tubes.

Table S1. Hydrodynamic radii and zeta potentials data of amino-functionalized silica particles in lanthanide chloride solutions at ionic strengths of 3 and 6 mM.

	3 mM		6 mM	
	Hydrodynamic radius	Zeta potential	Hydrodynamic radius	Zeta potential
	[nm]	[mV]	[nm]	[mV]
$LaCl_3$	162 ± 5	63 ± 0.8	170 ± 6	62 ± 1
$PrCl_3$	168 ± 2	56 ± 0.5	164 ± 2	61 ± 2
$SmCl_3$	175 ± 1	61 ± 2.3	169 ± 2	62 ± 2
$GdCl_3$	165 ± 2	64 ± 2.3	168 ± 2	58 ± 1
$DyCl_3$	178 ± 4	61 ± 1.1	167 ± 4	55 ± 1
$ErCl_3$	160 ± 0.1	57 ± 0.9	166 ± 1	65 ± 6
$TmCl_3$	163 ± 0.5	64 ± 1.1	163 ± 2	67 ± 1
$YbCl_3$	165 ± 0.6	59 ± 1.1	167 ± 1	64 ± 1
$LuCl_3$	161 ± 0.2	64 ± 1.1	163 ± 1	62 ± 0.3

Table S2. Nearest neighbor distance in non-close-packed structures of amino-functionalized silica particles dried from aqueous solutions of NaCl, $MgCl_2$, and $GdCl_3$ with ionic strengths of 0.1, 0.5, and 1 mM.

IS (mM)	NaCl	$MgCl_2$	$GdCl_3$
0.1	262 ± 25	284 ± 28	314 ± 31
0.5	229 ± 24	243 ± 23	257 ± 28
1	221 ± 25	230 ± 23	238 ± 23

Table S3. Hydrodynamic radii of amino-functionalized silica particles in NaCl, $MgCl_2$, and $GdCl_3$ solutions at ionic strengths ranging from 0.1 to 6 mM

IS (mM)	NaCl	$MgCl_2$	$GdCl_3$
0.1	169 ± 0.4	170 ± 3	172 ± 1
0.5	166 ± 2.3	171 ± 2	170 ± 1
1	169 ± 2.2	170 ± 2	168 ± 2
3	190 ± 2.0	172 ± 3	166 ± 2
6	250 ± 2.0	258 ± 5	165 ± 2

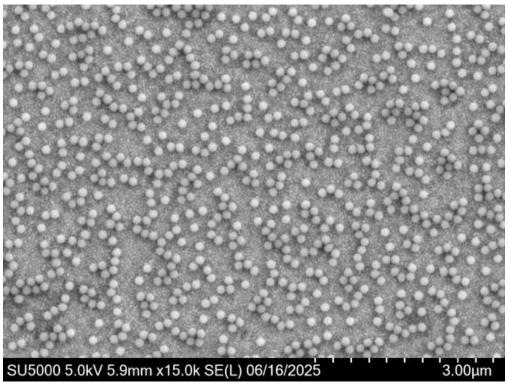

Figure S1. FESEM image of an array of amino-functionalized silica nanoparticles obtained by drying their dispersion in an aqueous $GdCl_3$ solution with an ionic strength of 600 mM on a gold surface.

References

(1) Barak, M.; Scharmann, P.; Nachtsheim, D.; Graf, C. Influence of Specific Ion Effects and Ion Valency on the Formation of Non-Close-Packed Monolayers of Silica Particles. *Colloids Surf. Physicochem. Eng. Asp.* **2025**, *720*, 137086. https://doi.org/10.1016/j.colsurfa.2025.137086.

(2) Hartlen, K. D.; Athanasopoulos, A. P. T.; Kitaev, V. Facile Preparation of Highly Monodisperse Small Silica Spheres (15 to >200 Nm) Suitable for Colloidal Templating and Formation of Ordered Arrays. *Langmuir* **2008**, *24* (5), 1714–1720. https://doi.org/10.1021/la7025285.

(3) Graf, C.; Gao, Q.; Schütz, I.; Noufele, C. N.; Ruan, W.; Posselt, U.; Korotianskiy, E.; Nordmeyer, D.; Rancan, F.; Hadam, S.; Vogt, A.; Lademann, J.; Haucke, V.; Rühl, E. Surface Functionalization of Silica Nanoparticles Supports Colloidal Stability in Physiological Media and Facilitates Internalization in Cells. *Langmuir* **2012**, *28* (20), 7598–7613. https://doi.org/10.1021/la204913t.

(4) Barak, M.; Aghdam, A. S.; Ozbulut, E. B. S.; Unal, S.; Cebeci, F. Ç. Layer-by-Layer Assembly of Nanofilms from Colloidally Stable Amine-Functionalized Silica Nanoparticles. *Colloids Surf. Physicochem. Eng. Asp.* **2021**, *630*, 127615. https://doi.org/10.1016/j.colsurfa.2021.127615.

(5) Schmudde, M.; Grunewald, C.; Risse, T.; Graf, C. Controlling the Interparticular Distances of Extended Non-Close-Packed Colloidal Monolayers. *Langmuir* **2020**, *36* (17), 4827–4834. https://doi.org/10.1021/acs.langmuir.0c00014.
